# The impacts of COVID-19 on K-8 science teaching and teachers

**DOI:** 10.1186/s43031-022-00060-3

**Published:** 2022-05-16

**Authors:** Meghan Macias, Ashley Iveland, Melissa Rego, Maya Salcido White

**Affiliations:** 1 University of California Santa Barbara, Santa Barbara, CA USA; 2 WestEd, San Francisco, CA USA

**Keywords:** Science instruction, COVID-19, K-8, Next generation science standards, Distance Learning, Survey Research

## Abstract

Some science education researchers have presented either isolated findings on specific points in time during the pandemic or non-empirical insights or suggestions for how teachers, district leaders, policymakers, and others should take up the learnings from the pandemic to move science education forward. However, there are few studies published to date that provide robust and longitudinal empirical data on what science instruction looked like throughout the pandemic and the magnitude of the impacts of the pandemic on science instruction when compared to pre-pandemic science teaching and learning. We conducted a primarily survey-based study on science instruction and enactment of the Next Generation Science Standards (NGSS) in K-8 classrooms throughout the COVID-19 pandemic. This analysis also incorporates a longitudinal dataset from grade 6–8 teachers across California on their NGSS instruction prior to and throughout the first year of the pandemic, providing insight on instruction over multiple years before and throughout distance learning. Our findings highlight the challenges that teachers and students faced during the pandemic, as well as the significant impacts that distance learning appeared to have on science instruction and teachers’ ability to provide NGSS-aligned instruction. However, we also found that a year after the initial school closures, teachers’ science instruction began to show improvements both in the frequency of science instruction (how often they were able to provide science instruction through distance learning) and the quality of science instruction (how often teachers were able to provide instruction that was aligned with the goals of the NGSS). Implications of this work are far reaching and may impact teachers, students, administrators, policymakers, professional learning providers, and curriculum developers regardless of whether science instruction occurs through distance learning or in-person moving forward.

## Introduction

The COVID-19 pandemic has had significant impacts on society in all sectors. In particular, educational systems have had to adapt to mostly virtual methods of instruction with school closures in effect for much of the last year in most of the United States. Online learning proved difficult to implement even before the pandemic made it essential, presenting challenges for students, educators, and staff (Gillett-Swan, 2017). However, it was especially difficult on the mass scale which occurred during the pandemic, and considering existing inequities in and across communities that left some students without adequate access to essentials like reliable internet, technological resources, or instructional materials (Holoquist et al., 2020; Morrar, 2020). Science education was no exception to widespread challenges faced during the COVID-19 pandemic and proved difficult to teach virtually from the beginning (Kurtz et al., 2020).

The Next Generation Science Standards (NGSS; NGSS Lead States, 2013) envision science learning as three-dimensional (3D), incorporating Science and Engineering Practices (SEPs), Crosscutting Concepts (CCCs), and Disciplinary Core Ideas (DCIs) in order to elicit student interest and engagement in science. The NGSS aim to engage students in these three dimensions of learning in a way that provides knowledge-rich experiences and deep learning, building on prior knowledge, experiences, and promoting student agency (Duschl & Bybee, 2014). This way of learning, however, was more challenging to implement during school closures in a virtual learning environment and with strict social distancing requirements in place (Authors, 2020) because it calls for student collaboration (which is not the same in a Zoom breakout room), in-depth hands-on investigations (for which materials are usually limited), and sophisticated teacher moves (that require more than just students to have their cameras on).

While some saw success with e-learning experiences or teaching science in a virtual environment (e.g., Babateen, 2011; Cavanaugh et al., 2004), the sudden move to distance learning due to the COVID-19 pandemic presented a myriad of issues for teachers, students, and other school officials in 2020 and 2021 (Kurtz, 2020; Wyse et al., 2020). For example, there was little time for the adaptation or adoption of science curricula that was designed for fully remote instruction across all grade levels (Marple & Le Fevre, 2021) and students and teachers alike were battling a profound sense of isolation and lack of community during quarantine that left them without critical socioemotional supports (Carnegie Math Pathways, 2021; Folsom, 2021). This radically different teaching and learning context highlighted the need to investigate how science was taught throughout the pandemic and the impacts on instruction and students’ opportunities to engage in NGSS-aligned science. Furthermore, the lessons learned during this time period should be carried into schools as they return to in-person instruction.

### Literature review

This study builds on the existing literature around teaching during unanticipated distressing events such as an epidemic, a natural disaster, or a school shooting (e.g., Liu et al., 2012; Lee, 1999; Tsai, 2001; Prinstein et al., 1996; Le Brocque et al., 2017; Schiller, 2013; Wike & Fraser, 2009). However, more than a year after widespread distance learning began, there is as of yet little empirical research on what occurred during distance learning and how lessons learned during this period should inform science education more broadly moving forward. Currently, anecdotal documentation of science teaching and learning during COVID-19 has become available. Much of this body of literature has found that given the right support and appropriate amount of time, teachers can be successful at engaging students in meaningful online learning experiences (e.g., Ames et al., 2021; Rannastu-Avalos & Siiman, 2020; Rouleau et al., 2021; Schwartz et al., 2020). However, many of these studies were largely anecdotal, had small sample sizes, were primarily qualitative in nature, and do not include longer-term data through spring 2021 when many students were still online (Campell et a. 2021; Darling Hammond & Hyler, 2020). Further empirical research needs to be done that uses larger sample sizes, draws on mixed methods approaches, and draws on a longer-term data set across the duration of the pandemic. This study addresses gaps in the literature, ultimately working to inform both distance and in-person science teaching and learning moving forward. This work provides valuable insight on what education systems can do to provide quality science instruction and appropriate teacher support during times of crisis.

### Theoretical framework

While other research largely focuses on a single element or context within the education system during the COVID-19 pandemic, we posit that research must take a broader view when investigating the impacts of the pandemic. For this study we drew on and adapted the idea of concentric circles emanating out from the learner within the center from theories like Bronfenbrenner’s ecological systems model (1979). Our adapted model considered each district as its own system, with smaller, nested systems within it (the school and classroom). Typically, within the classroom is the learner, which includes both the student and the teacher (Lieberman, 1995). We acknowledge that these systems are not cleanly nested, and networks between, across, or outside of them may affect learners within (Lave & Wenger, 1991; Wenger, 1998; Wilson, 1993). This perspective views learning as contextual and social and influenced by factors occurring within and outside of these systems (Peressini et al., 2004).

As such, this view lends itself to investigations of students and teachers in an education system that was rattled by the COVID-19 pandemic. In other words, we adopt Bronfenbrenner’s ecological model to investigate the impacts of the COVID-19 pandemic on a complex, layered system. Investigating science instruction through this lens better accounts for the fact that the implementation of instructional changes or improvements can only occur when the many components within the education system are in alignment (Century & Cassata, 2016). We sought to understand how the pandemic impacted these components within the education system, how they changed over time, and whether or to what extent these changes affected science instruction in particular. Within this larger system, we focus on science instruction from the teachers’ perspective, but gain their insights on other components of the education system such as district policies, supports, and student engagement, which influence the teacher and their ability to enact high-quality science instruction.

### Research questions

This study examined the effects of school closures caused by the COVID-19 pandemic on K-8 science instruction. This study was guided by the following research questions:

During the COVID-19 pandemic:

1. What opportunities and challenges were teachers of science experiencing?
2. What tools, resources, and supports were teachers of science receiving and utilizing to guide their science instruction?
3. What elements of the NGSS were teachers addressing and how was science being enacted in their classes?

## Methods

The research presented below is from analyses done on two national surveys on the impacts of COVID-19 conducted during the spring/summer of 2020 and the spring of 2021, as well as surveys from a smaller subsample of California grade 6–8 teachers from an ongoing study of the NGSS.

### Context and participants

This study was part of a multi-year project with over 100 grade 6–8 public school teacher participants across California (“NGSS Study”). In late spring of 2020, the study team developed and distributed a survey about NGSS distance learning instruction during COVID-19 related school closures nationally, with a follow-up survey that was distributed in the spring of 2021. Participants included a total of 342 teachers in 2020 representing 25 states, and 193 teachers in 2021 from 15 states, with California and New Mexico consistently represented the most. Participants had to be K-8 teachers who were teaching science prior to the shift to distance learning. Participants were primarily upper elementary and middle grades teachers, with many who were grade 6–8 teachers in California who had also responded to at least one prior survey as a part of the multi-year NGSS study. Teachers were recruited through emails to professional organizations, social media posts, and by contacting district personnel.

### Data collection

Researchers developed a survey instrument based on several existing valid and reliable surveys widely used in science education research (e.g., Bae et al., 2016; Banilower et al., 2018; Enochs & Riggs, 1990; Lumpe et al., 2000; Reiser et al., 2017). End-of-year (EOY) teacher surveys documented participants’ overall impressions of the aspects of the NGSS they taught throughout the year. These surveys were completed in the summer of 2018 and 2019; two surveys were disseminated in early spring 2020 (asking teachers to reflect on their practice prior to school closures due to COVI19), and summer 2020 (asking teachers to reflect on their practice during school closures). Finally, the last survey was disseminated in spring 2021. Surveys solicited participants’ retrospective views of teaching and learning during the prior school year (as they were given at the end of the year in the spring/summer) with the exception of the second 2020 survey which asked teachers to reflect specifically on teaching and learning during the semester of school closures. Pre-COVID surveys (summer 2018, 2019, and spring 2020) included 131 total closed-ended survey questions. Pre-COVID surveys included questions about NGSS implementation (e.g., thinking about your science instruction over the past year, how often did you incorporate asking questions?), the role of phenomena in instruction (e.g., thinking about your science instruction over the past year, to what extent were you able to use science and/or engineering phenomena as a substantial driver of instruction?), as well as items related to equity (e.g., thinking about your science instruction over the past year, how often did you incorporate students’ cultural backgrounds into science instruction?).

In May 2020, researchers developed the “Distance Learning survey” which asked many questions that were on the EOY survey, but also drew on new surveys specific to distance learning during COVID-19 (e.g., Kurtz, 2020) and included 140 multiple choice, Likert-scale, and open-ended questions. The survey included questions about teachers’ contexts (e.g., school district, teaching responsibilities pre- and during pandemic), change in levels of student learning and engagement before and during the pandemic, alignment of distance learning with the NGSS, and support for distance learning from outside sources (e.g., district or school-level support). See supplementary materials for full surveys. This survey was distributed nationwide in Summer 2020 and Spring 2021, and to participants in the NGSS study. See Table 1 below for a timeline of when the surveys were disseminated, who participated, and the number of participants.

**Table 1:** Time and year or survey dissemination, total number of participants, and sample demographics by year

Survey time/year	N	Participant demographics by year
Summer 2018	119	NGSS study participants: middle school science teachers from California
Summer 2019	109	NGSS study participants: middle school science teachers from California
Spring 2020*	120	NGSS study participants: middle school science teachers from California
Summer 2020*	452	NGSS study participants: middle school science teachers from California + expanded sample: K-8 science teachers across US
Spring 2021	193	NGSS study participants from California + expanded sample: K-8 science teachers across US

*Spring 2020 survey asked about in-person instruction prior to school closures. Summer 2020 survey asked about distance-learning instruction during school closures

Insert Table 1 about here.

### Data analysis

First, the Distance Learning survey responses were examined for all respondents. Multiple choice survey items were analyzed descriptively (e.g., frequencies were run, and percentages were computed) and statistical t-tests and analysis of variance (ANOVAs) with post-hoc tests were performed to determine statistically significant differences over time (between spring 2020 and spring 2021) as well as between specific groups (e.g., grade K-2 vs. 3–5 vs. 6–8 teachers). Open-ended survey items were analyzed using emergent coding methods (Saldaña, 2011) to uncover themes in teacher responses. Next, researchers compared the Distance Learning survey responses from the grade 6–8 teachers in California to the responses of similar teachers from prior years’ surveys. This allowed researchers to look at changes over time of NGSS implementation before and during the COVID-19 pandemic among this subsample of California science middle grades teachers.

## Results

### Science learning and engagement

**Fig. 1:**
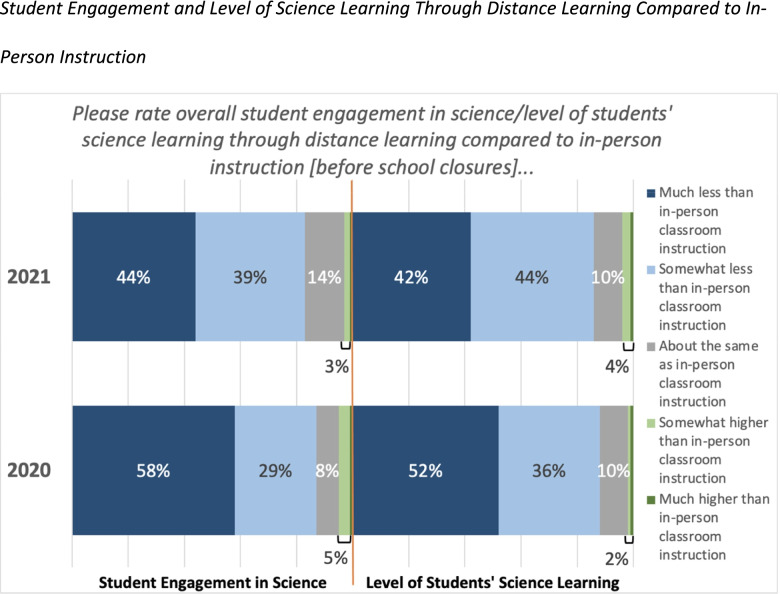
Student Engagement and Level of Science Learning Through Distance Learning Compared to In-Person Instruction

Our survey found that when teachers were asked to compare student engagement during school closures in spring of 2020 and spring of 2021 to student engagement through in-person learning before school closures, they reported much less student learning and engagement throughout the pandemic. As shown in Fig. 1, more than half of respondents in spring of 2020 reported that students were engaged “much less” through distance learning when compared to regular classroom instruction before school closures. The following year in spring of 2021, data showed a subtle improvement, with slightly fewer teachers (44%) reporting that students were engaged “much less” through distance learning compared to before school closures. Furthermore, a majority of teachers in spring of 2020 (52%) reported that students were also learning less science during school closures when compared to before school closures, but this finding also had similar gains by spring of 2021.

When disaggregated by grade band, results of the ANOVA indicated that grade 6–8 teachers indicated that students in their classes were significantly less engaged (F(2, 261) = 6.68, *p* = 0.0015) than those in the elementary grades, and that the youngest students (K-2) were the most engaged overall. This may show that there were challenges for science education at the secondary level that were impacting students’ learning and engagement in science instruction in an online format.

### Time spent on science

**Fig. 2:**
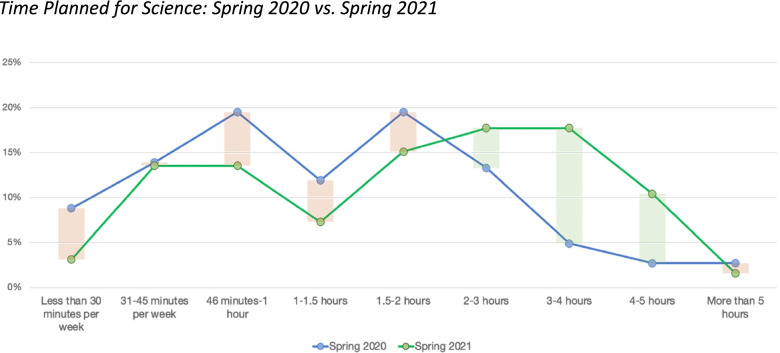
Time Planned for Science: Spring 2020 vs. Spring 2021

Another challenge of online teaching was that teachers were spending overall less time with students and thus spending less time on science. In the spring of 2020, most teachers (88%) indicated that students were spending less time on science through distance learning, with teachers saying that they planned between 1 and 2 hours of science instruction per week on average. However, by the spring of 2021 teachers indicated spending more time on science, planning upwards of 3–5 hours per week on average (see Fig. 2). While this shows how much time teachers *planned* for students to spend and does not necessarily reflect the *actual* amount of time students were engaged in science learning, these findings still show that teachers were able to incorporate more science time into their instruction by the spring of 2021.

Overall, teachers reported that there was substantially less time spent on science during school closures, but by spring of 2021 teachers had persisted and were starting to incorporate more science, more often. However, time spent on science does not reflect the *quality* of science instruction that students were receiving. Below, we discuss whether students had opportunities to engage in rigorous, NGSS-aligned science learning during school closures.

### NGSS-aligned instructional methods

**Fig. 3:**
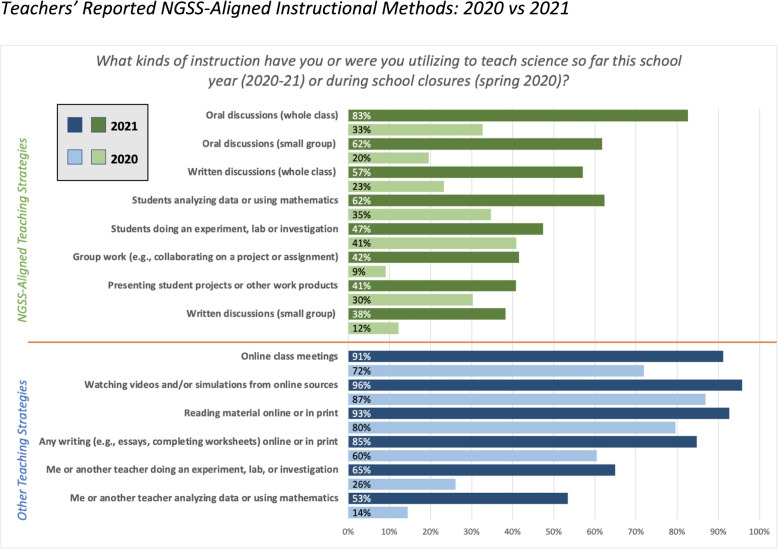
Teachers’ Reported NGSS-Aligned Instructional Methods: 2020 vs 2021

Figure 3 below shows that early in the pandemic in spring of 2020 teachers overwhelmingly relied on teaching strategies that were not aligned with the goals of the NGSS, such as watching videos or online simulations and reading material with very little implementation of investigations, discussions, group work, or analyzing data in ways that would promote student agency and deeper science learning. There were some changes in Spring 2021; the strategies that are not NGSS aligned were not used by as many teachers overall, and there were dramatic increases in many of the strategies that correlate to NGSS-aligned instruction that would further student science learning.

For example, a key element of NGSS instruction, investigations, had some drastic changes occur during the pandemic. While in 2020 nearly twice as many teachers said that the students were doing investigations (41%) as opposed to the teachers (26%), by spring 2021 much more teachers reported doing the investigations themselves at least “sometimes” (65%), which is significantly more than the 47% who said students were doing the investigations at least “sometimes”. This means that investigations actually became *less* NGSS-aligned after spring 2020 because teachers were not having their students engage in the investigations themselves, limiting their agency and ability to fully engage in the “figuring out” of phenomena that is a main goal of the NGSS.

Student discourse also increased dramatically from spring 2020 to spring 2021, seeing two- and three-fold increases in the number of teachers who engaged their students in both oral and written discussions in whole class and small group context between these time points. In addition, more teachers had their students engage in group work (albeit virtually) with only 9% of teachers saying they had students do any group work in spring 2020, to 42% of teachers having their students do group work at least “sometimes” during the 2020–21 school year.

We took a closer look at what was happening during distance learning in 2021, broken down by grade band. When we ran ANOVAs across grade bands (K-2, 3–5, or 6–8), drawing on the same survey questions about NGSS-aligned instructional methods, we found some statistically significant differences. On average, middle school teachers (grades 6–8) were more likely than K-2 or 3–5 teachers to engage students in group work (F(2, 258) = 5.45, *p* = 0.005) and in written (F(2, 259) = 7.73, *p* < 0.001) and oral discussions (F(2, 258) = 7.08, *p* = 0.001), and these differences were statistically significant. K-2 and 3–5 teachers were more likely than middle school teachers to use videos and engage students in reading material online or in print, although these differences were not statistically significant.

**Fig. 4:**
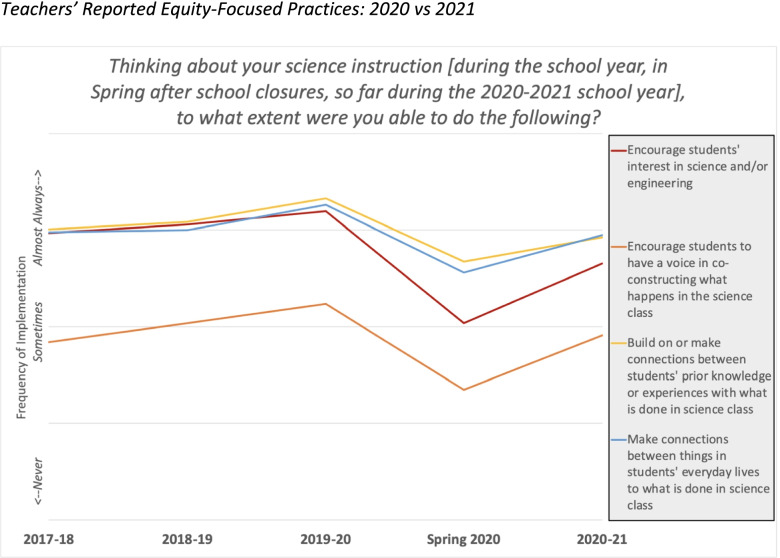
Teachers’ Reported Equity-Focused Practices: 2020 vs 2021

Next, we were able to examine longitudinal differences in NGSS-aligned instruction amongst the NGSS study, California middle school science teacher sample. We analyzed survey questions that asked about the frequency of implementation of NGSS-aligned instructional methods that are associated with equity and diversity. When we asked to what extent grade 6–8 teachers in California were able to encourage student interest in science, encourage student voice in co-constructing what happens in science class, build on students’ prior knowledge or experiences, and make connections to students’ everyday lives, teachers reported a sharp decrease in 2020 compared to previous years (e.g., 2018 and 2019), with an upward trend by spring 2021 (Fig. 4). All of these instructional strategies are those which not only encourage NGSS-aligned science instruction, but also promote more equitable engagement in deep science learning.

### NGSS-aligned instruction

When asked how difficult it was to implement the SEPs, 60% of teachers reported that it was “much more” difficult to implement this feature of the NGSS in 2020 than prior to school closures. When asked the same question in 2021, teachers reported that this was slightly less difficult than in spring of 2020, though still “somewhat more” difficult compared to before school closures. This trend held for the CCCs as well. See Table 2 to see the mean increases for all SEPs and CCCs teachers reported incorporating into their instruction for 2020 and 2021. These increases from 2020 to 2021 were all statistically significant.

**Table 2:** T-test Results of Teachers Reported Incorporation of SEPs and CCCs in 2020 and 2021

	Sig	t	df	Mean Difference
Change in asking questions between 2020 and 2021	0.01	−5.65	419.72	0.53
Change in planning investigations or experiments between 2020 and 2021	0.01	−2.34	458.50	0.23
Change in doing investigations between 2020 and 2021	0.002	−2.10	449.04	0.28
Change in coming up with explanations between 2020 and 2021	0.001	−4.47	446.86	0.40
Change in developing and using models between 2020 and 2021	0.001	−4.46	446.86	0.40
Change in using evidence to support a claim between 2020 and 2021	0.001	−5.27	431.17	0.54
Change in recording observations between 2020 and 2021	0.001	−3.82	383.98	0.42
Change in evaluating information between 2020 and 2021	0.001	−3.12	440.54	0.29
Change in graphing data between 2020 and 2021	0.001	−5.06	392.95	0.50
Change in analyzing and interpreting data between 2020 and 2021	0.002	−3.17	410.46	0.33
Change in looking for patterns in data between 2020 and 2021	0.001	−5.57	408.55	0.58
Change in designing the steps needed to answer questions between 2020 and 2021	0.001	−4.16	419.82	0.41
Change in cause and effect between 2020 and 2021	0.001	−3.45	383.10	0.24
Change in patterns between 2020 and 2021	0.001	−4.06	373.68	0.43
Change in scale, proportion, and quantity between 2020 and 2021	0.008	−2.67	352.53	0.37
Change in systems and system models between 2020 and 2021	0.001	−4.32	393.23	0.50
Change in energy and matter between 2020 and 2021	0.001	−4.32	386.49	0.47
Change in structure and function between 2020 and 2021	0.001	−4.59	413.25	0.26
Change in stability and change between 2020 and 2021	0.001	−2.49	402.22	0.20

*Note:* Scale is 0 = not at all, 3 = somewhat, 5 = to a great extent.

Though it seemed to get slightly easier for teachers to engage their students in the SEPs through distance learning over time, findings still indicate that SEPs were more challenging to implement during school closures than prior to the pandemic. This increase from 2020 to 2021 was good, but the status reported in spring 2021 may not reflect the full magnitude of the impacts on science instruction that distance learning had.

**Fig. 5:**
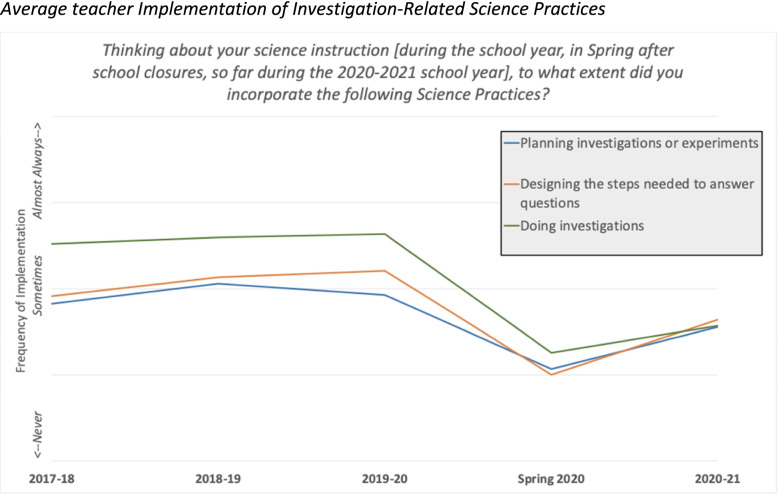
Average teacher Implementation of Investigation-Related Science Practices

A look at longitudinal data from our subsample of California middle school science teachers reveal much more dramatic decreases in SEP and CCC implementation during the pandemic. For example, Fig. 5 shows a significant decrease in reported implementation of practices related to planning and conducting investigations in spring of 2020 compared to previous years, and then a marked increase in 2021. However, while our spring 2021 survey showed that on average teachers were incorporating all practices more often, the frequency is still far below pre-pandemic levels.

When examining differences by grade level, we found some notable differences in their reported SEP and CCC implementation. K-2 teachers were more likely than middle school teachers to report both planning (F(2, 258) = 4.71, *p* = 0.009) and doing investigations (F(2, 258) = 4.10, *p* = 0.01) more often, and this difference was statistically significant. However, middle school teachers were more likely than K-2 and 3–5 teachers to report developing and using models (F(2, 258) = 7.52, *p* < 0.001), using evidence to support a claim (F(2, 256) = 14.53, p < 0.001), evaluating information (F(2, 258) = 5.44, *p* = 0.004), recording observations (F(2, 257) = 6.74, *p* = 0.001), graphing data (F(2, 256) = 5.87, *p* = 0.01), and analyzing and interpreting data (F(2, 256) = 15.07, *p* < 0.001) more often. This suggests that while younger students were engaged in investigations, they were less likely to have opportunities to engage in other important pieces of NGSS-aligned science.

### Evolving challenges

The primary challenges that K-8 teachers cited most often throughout the pandemic stayed relatively constant (see Table 3). However, teachers of grade 6–8 students felt the lack of hands-on investigations and low levels of student participation, motivation, and engagement were more challenging than for elementary teachers, while elementary teachers felt the lack of collaboration and discourse and lack of science materials and supplies for students were more challenging. All teachers cited equity issues that impacted instruction in spring 2020. Despite knowing that instruction was very likely to be and remain virtual, these most cited challenges largely stayed constant from spring 2020 through spring 2021. There were some changes over time, however. For example, low student participation, motivation, and engagement were rated as a major challenge in the early days of the pandemic, but then were replaced by challenges related to student collaboration and discourse by spring 2021.

**Table 3:** Challenges to Teaching Science in K-8 Through Distance Learning: 2020 vs. 2021

What have you found to be the most challenging about implementing successful distance learning for science specifically?	Spring 2020	Spring 2021
Less hands-on, inquiry, and exploration/investigation	1st (44%)	1st (88%)
Low student participation, motivation, and engagement in science online.	2nd (34%)	4/5th (55%)
Lack of science materials and supplies for students	3rd (24%)	4/5th (55%)
Less student collaboration/discourse	4th (16%)	2nd (76%)
Issues students face using technology (internet, devices, platforms, skill and/or experience with technology)	5th (15%)	3rd (59%)

*Note:* This question was an open-response in spring 2020, and responses were open-coded. The themes uncovered in this coding led to the response options that were provided to teachers in the spring 2021 survey for this item. Therefore all responses are reported by how often teachers cited that challenge within each survey/year and cannot be readily compared across years

Insert Table 3 about here.

Overall, teachers at the beginning of the pandemic struggled to get students to participate in online learning, with students often failing to attend synchronous lessons, and not participating or engaging in instruction even when they were there. Many of these issues were less challenging in 2021, with the elementary grades K-5 no longer citing this as one of their top five challenges. This change may be attributed to having more time with students, many people putting in much work to ensure that *all* students are now able to join online meetings using provided technology, students and teachers both being supported more across the board, and teachers utilizing more and different ways to engage students in this virtual format.

The challenge with a lack of materials that teachers and students had access to was not limited to those needed for investigations. Teachers’ lack of access to curriculum materials that were appropriate for distance or hybrid learning environments was also a common challenge during school closures. Prior to the pandemic, teachers reported using commercially published kits and modules; state, district, or school-developed units and lessons; or other commercially developed online and print materials in their science lessons. After shifting to online instruction, teachers reported utilizing materials they created on their own or with colleagues, or lessons and resources from free online sources. Many teachers also indicated needing to draw from multiple resources to have materials that worked for their online classroom. This suggests that teachers put together their own sets of materials for remote instruction, as they lacked materials that were designed for distance learning. Teachers who were in districts that had already adopted NGSS-aligned curricula prior to the pandemic reported using these materials they had in whatever way they could.

### Opportunities for science education during COVID

Our findings indicate that there were statistically significant increases in some key features of NGSS-aligned instruction. For example, as mentioned earlier, there were significant increases in SEP and CCC implementation in 2021 when compared with their implementation in 2020. Furthermore, we found that there were also increases in how often teachers were able to encourage student agency in science and in how often they connected science instruction to students’ everyday lives, prior knowledge, or experiences when compared 2020 reports (Table 4). Encouraging student agency in the class (e.g., giving students ownership in their science learning) was statistically significant. Importantly, teachers reported a significant increase in providing mental and emotional support to students in 2021 when compared to 2020.

**Table 4:** T-test Results of Teachers Reported Incorporation of Student Agency, Prior Experiences, and Mental/Emotional Health Between 2020 and 2021

	Sig	t	df	Mean Difference
Change in giving students ownership in their science learning between 2020 and 2021	0.001	3.73	440.87	0.33
Change in supporting students mentally and emotionally between 2020 and 2021	0.001	−4.05	322.72	0.56
Change in relating science instruction to students’ home lives or communities 2020 and 2021	0.99	0.01	322.72	0.0018

*Note:* Scale is 0 = not at all, 3 = somewhat, 5 = to a great extent.

Insert Table 4 about here.

By 2021, teachers were utilizing teaching methods that encouraged student voice, built on students’ prior knowledge or experiences, and made connections to students’ everyday lives’, as well as attending to student mental and emotional health. This increase indicates a return to the quality of instruction happening pre-pandemic.

In addition to these important opportunities and in contrast to most of our survey respondents, a small group of teachers reported increased student engagement and learning during distance learning. The teachers who felt their students were more engaged in science during distance learning when compared with in-person instruction cited the following reasons for this change: capitalizing on flexible schedules; using familiar low-cost or no-cost materials to engage students in science; and encouraging student ownership of their new learning environments. In open-ended survey questions, teachers elaborated on why they thought their students were more engaged. Several teachers offered that the more relaxed schedule and the removal of hard deadlines allowed students to spend more time engaged with science phenomena in their classrooms. In addition, some teachers reported feeling that they had more time to teach science because state tests were suspended during the pandemic and so there was less pressure to focus on ELA and math for testing purposes.

## Discussion

As the research presented here shows, many teachers reported drastic reductions in high-quality science in their classrooms and districts as a result of the pandemic. Elementary teachers in particular seemed to be able to implement science instruction more often, perhaps because they were able to fold science into their ELA and math lessons (Pesnell, 2020). This finding differs from previous literature that has historically shown However, just because elementary teachers were able to do more science instruction, it does not necessarily follow that they were doing more NGSS science instruction. As our findings show, elementary teachers were more likely to do investigations with their students, but less likely to implement other SEPs when compared to middle school teachers. This could be due to elementary teachers’ generally low self-efficacy to teach science (National Academies of Sciences, Engineering, and Medicine, 2021). This finding suggests a need for NGSS-aligned curriculum that not only is adaptable for distance learning, but is also educative in its ability to support teachers to provide NGSS-aligned science instruction and engage their students in deep science learning.

Additionally, our findings showed that elementary teachers were less likely to engage students in group work and written and oral discussions. This may be because engaging in group work and written discussions, in particular, through distance learning most often takes the form of using breakout rooms, having students coordinate and meet independently, or type written responses, all of which require more technology fluency and digital literacy than many elementary school students have. This finding aligns with other similar studies of K-8 distance learning which have found that teachers relied more on practices that gave students interaction with content and to the teacher, but rarely with their peers (Kara et al., 2022). In addition, oral discussions online with young students can be especially difficult because the teacher cannot easily discern what is being said if multiple students are talking at once, or if there is a noisy background in the students’ learning environment. This highlights the two-pronged requirement of science education during distance learning: students and teachers not only need to acquire scientific literacy but digital literacy as well.

However, elementary teachers were not the only teachers struggling to teach science via distance learning. After schools initially closed, teachers at all levels struggled to keep students engaged in high quality science lessons and they reported less science learning throughout the whole first year of the pandemic. As our survey findings showed, investigations actually became *less* NGSS-aligned after spring 2020 because teachers were the ones conducting investigations, not students. This makes sense given that teachers struggled to receive and distribute supplies to students for investigations through distance learning (Authors, 2020, 2021). Furthermore, teachers were burdened with not only learning how to teach virtually, but also had to become their own curriculum developers, spending a great deal of time searching for and compiling instructional materials that suited their needs and the needs of their students. With materials and lessons coming from so many different sources, it is to be expected that lessons and units were not as coherent or NGSS-aligned as they normally would be. However, this presents a challenge for student learning and engagement. The numerous challenges presented above, including technology issues as well as fewer hands-on, investigation experiences, may shed light on why teachers reported less student learning and engagement. This aligns with other research that has found that science, in particular, was the most difficult to teach virtually (Kurtz, 2020). However, over time, some of these challenges were at least somewhat resolved.

The difference between 2021 and 2020 responses indicated that teachers were beginning to adapt to new modes (e.g., online class meetings) and methods (e.g., discussions using chat or message boards) of instruction. These general increases seen in 2021 are likely attributable to teachers’ resiliency and dedication to teaching NGSS-aligned science despite having to surmount huge barriers in doing so. As reported in our findings, some teachers that experienced success in 2021 capitalized on flexible schedules, an important practice to create more manageable workloads for students, as identified by prior studies (Johnson, 2021). Importantly, this is no small finding; teachers continued resilience to support their students (e.g., increasing emotional support provided reported in our surveys) is a powerful example and reminder of the important work that teachers do (Lowenhaupt et al., 2021). When specifically examining NGSS implementation though, most teachers still reported that learning and engagement remained much lower throughout school closures than with in-person instruction prior to the pandemic. In short, the rebound in the quality of NGSS-aligned science instruction still fell short of pre-pandemic levels.

## Conclusions

The work presented here provides important information about the state of K-8 science education during distance learning. While hopefully a once in a lifetime event, the COVID-19 pandemic has forced us as a society to confront the need for scientifically literate citizens and the need for high-quality experiences for all students. The pandemic makes it clearer than ever the pressing need for the critical skill to engage in scientific discussions. This makes the case for high quality science education at all educational levels even more salient; knowing the level and quality of science education over the past 2 yrs informs what steps we need to take next. COVID-19 hit just when many districts were beginning to implement the NGSS, sharply reducing the amount and quality of science taught. The need for NGSS-aligned instruction has never been greater. Furthermore, since periodic shifts to distance learning or hybrid learning continue to be a practice in many areas of the U.S. that continue to battle the COVID-19 pandemic, we will likely see some similar challenges to what we found here with distance learning. This makes this research even more crucial as we all continue to navigate the ongoing COVID-19 pandemic.
